# Application of Bayesian networks to GAW20 genetic and blood lipid data

**DOI:** 10.1186/s12919-018-0116-y

**Published:** 2018-09-17

**Authors:** Richard A. J. Howey, Heather J. Cordell

**Affiliations:** Institute of Genetic Medicine, Newcastle University, Central Parkway, Newcastle upon Tyne, NE1 3BZ UK

## Abstract

**Background:**

Bayesian networks have been proposed as a way to identify possible causal relationships between measured variables based on their conditional dependencies and independencies. We explored the use of Bayesian network analyses applied to the GAW20 data to identify possible causal relationships between differential methylation of cytosine-phosphate-guanine dinucleotides (CpGs), single-nucleotide polymorphisms (SNPs), and blood lipid trait (triglycerides [TGs]).

**Methods:**

After initial exploratory linear regression analyses, 2 Bayesian networks analyses were performed. First, we used the real data and modeled the effects of 4 CpGs previously found to be associated with TGs in the Genetics of Lipid Lowering Drugs and Diet Network Study (GOLDN). Second, we used the simulated data and modeled the effect of a fictional lipid modifying drug with 5 known causal SNPs and 5 corresponding CpGs.

**Results:**

In the real data we show that relationships are present between the CpGs, TGs, and other variables—age, sex, and center. In the simulated data, we show, using linear regression, that no CpGs and only 1 SNP were associated with a change in TG levels, and, using Bayesian network analysis, that relationships are present between the change in TG levels and most SNPs, but not with CpGs.

**Conclusions:**

Even when the causal relationships between variables are known, as with the simulated data, if the relationships are not strong then it is challenging to reproduce them in a Bayesian network.

## Background

Genome-wide association studies (GWAS) have been very successful at detecting genetic variants (typically single-nucleotide polymorphisms [SNPs]) associated with phenotypic outcomes. A typical approach to understanding the identified relationships between phenotype and associated genetic factors is to use public databases to see if the observed association can be explained by gene expression or DNA methylation patterns in tissue types relevant to the phenotype in question. However, public databases contain measurements made in different individuals compared to those used in the GWAS analysis, possibly even measured a different species. Consequently, there is interest in using causal inference methods applied to measurements of potential intermediate variables (such as gene expression or DNA methylation) taken in the *same* set of individuals as are included in the GWAS data set, allowing more direct conclusions about causality to be made. With the increase in different data types comes the desire to model more complex causal relationships beyond using just 2 or 3 variables at a time. This is possible with the use of Bayesian networks, where many variables can be modeled simultaneously in an exploratory manner, providing a natural extension to 3-variable causal modeling. In a recent study, Ainsworth et al. [1] compared Bayesian networks with other causal inference methods in the 3-variable situation, and found the Bayesian networks to perform competitively. We here attempt to gain insight into the conditional dependencies between the variables in the GAW20 data set by fitting Bayesian networks (separately) to the GAW20 real and simulated data. The GAW20 real data are based on a previous study into the association between differential methylation of cytosine-phosphate-guanine dinucleotides (CpGs) and the blood lipid trait, triglycerides (TGs), which study found a region of the epigenome with 4 CpGs significantly associated with TGs. The GAW20 simulated data model the effect of a fictional drug that affects TGs via both SNP and CpG effects, with methylation of the corresponding CpG site modifying the effect of the SNP on TG levels. These analyses were performed with knowledge of the GAW20 “answers.”

## Methods

### Real data

The GAW20 real data [2] consisted of phenotype and covariate data before and after fenofibrate drug treatment for 3 weeks. Individuals had measurements taken at 4 visits: visits 1 and 2 before treatment and visits 3 and 4 after treatment. Methylation measurements on CpGs were taken at visits 2 and 4. In the Genetics of Lipid Lowering Drugs and Diet Network (GOLDN) study on which the GAW20 data was based, Irvin et al. [3] performed an epigenome-wide association study (EWAS) and found 4 CpGs in the same region of the epigenome that were significantly associated with TGs.

We performed a similar EWAS to show that these 4 CpGs are significantly associated with TGs in the GAW20 data at visits 2 and 4. From a total of 1105 individuals, 995 had methylation data at visit 2 and 530 at visit 4. We used linear regression of the logged TG levels (as TGs are approximately log-normally distributed), and included covariates for the age, sex, and center (Minneapolis or Salt Lake City):

$$ \log (TG)={\beta}_0+{\beta}_1 CPG+{\beta}_2 age+{\beta}_3{I}_1(center)+{\beta}_4{I}_2(sex)+{\beta}_5 pc1+{\beta}_6 pc2+{\beta}_7 pc3+{\beta}_8 pc4+\epsilon $$

where *CPG* is the methylation of the CpG being tested and *ϵ* is a random error. The *β*_*i*_s are regression coefficients and *I*_*j*_s are indicator functions for the two discrete variables. We included 4 principal components based on the methylation data to account for potential biases such as batch effects. We used the R software package [4] to perform the tests, and did not account for family structure (relatedness between individuals) as obtaining accurate *P* values for discovery was not the main aim of our analysis.

We then used the data from the 4 CpGs that we and Irvin et al. [3] found to be significantly associated with TGs to fit a Bayesian network. We used the CpG data taken at visit 2 (as this had a larger number of measurements than data taken at visit 4) and data on age, sex, and center. Following preliminary GWAS analysis between SNPs and CpGs, and between SNPs and logged TG levels, we did not find any convincing associations; consequently, we did not include any SNPs in our Bayesian network analysis. No CpGs at visit 2 (or visit 4) were associated with change in TG levels as a result of drug treatment, so, in contrast to the GAW simulated data analysis (described later), we did not fit a Bayesian network modeling change in TG levels (ie, TG levels after treatment, with TG levels before treatment included as a covariate) as an outcome.

We implemented the Bayesian network method given by Scutari and Denis [5], which was chosen as being the most appropriate for mixed discrete and continuous data. We used our own C++ implementation, BayesNetty [6], with a hill-climbing algorithm, random restarts, and the Bayesian information criterion for model selection. Categorical variables, sex and center, are automatically constrained to have no parents in the Bayesian network analysis. An “average network” was also calculated by finding the best-fit model 1000 times using bootstrapped data. The *strength* of an edge was then given by the proportion of networks where it was present in either direction. The *direction* of the edge was given by the proportion of times it was in a given direction when present. The average network provides an estimate of the direction of causality between variables. A strength threshold was applied to network when it was plotted so that only edges that are considered of interest are plotted. The networks were drawn using the igraph [7] R package.

### Simulated data

The GAW20 simulated data was designed to model the effect of a fictional drug on TG levels. The data was only simulated for visits 3 and 4, with the real data at visits 1 and 2 forming the basis for the simulated data. We viewed the documentation for the simulation that indicated there were 5 causal SNPs, each with one nearby corresponding CpG, that were used to simulate change in TG levels between drug treatments. The simulation method used CpG data at visit 4 to determine the change in TG levels; consequently, we chose to use visit 4 CpG data in our analyses. We analyzed simulated data replicate number 84 as suggested by the GAW20 organizers as the best representative replicate.

For our analysis, the SNP data was restricted to SNPs with a minor allele frequency greater than 0.01 and the CpG data was left unmodified. We attempted to find SNPs associated with outcome using FaST-LMM (Factored Spectrally Transformed Linear Mixed Model) [8] to account for family structure via the following mixed model:

$$ \log (TG4)={\beta}_0+{\beta}_1 TG2+{\beta}_2 SNP+{\beta}_3 age+{\beta}_4{I}_1(center)+{\beta}_5{I}_2(sex)+\epsilon $$

where *ϵ* is the random error, structured to account for estimated relatedness, the *β*_*i*_s are regression coefficients and *I*_*j*_s are indicator functions for the two discrete variables. The TG levels at visits 2 and 4 are given by *TG2* and *TG4*. By including *TG2* as a covariate, we effectively test for association with the change in TG levels between visits 2 and 4. The SNP data, *SNP*, are given by the number of minor alleles, 0, 1, or 2.

An EWAS to detect CpGs associated with the change in TG levels was also performed as follows:

$$ {\displaystyle \begin{array}{c}\log (TG4)={\beta}_0+{\beta}_1 TG2+{\beta}_2 CPG4+{\beta}_3 age+{\beta}_4{I}_1(center)+{\beta}_5{I}_2(sex)\\ {}+{\beta}_6 pc1+{\beta}_7 pc2+{\beta}_8 pc3+{\beta}_9 pc4+\epsilon \end{array}} $$

where *CPG4* is the CpG level at visit 4 and other coefficients and variables are as previously. A Bayesian network was fitted to the 5 causal SNPs and the 5 causal CpGs together with variables for age, sex, center, and TG levels at visits 2 and 4. We obtained the best-fit network as well as calculating an average network using the same methods as before. The fitting of the Bayesian networks was constrained such that *TG2* was a parent of *TG4*. With this constraint, the change in TG levels between visits 2 and 4 can be modeled. Also, SNPs were constrained to have no parents and CpG data at visit 4 could not be parents of *TG2*.

## Results

### Real data

**Fig. 1:**
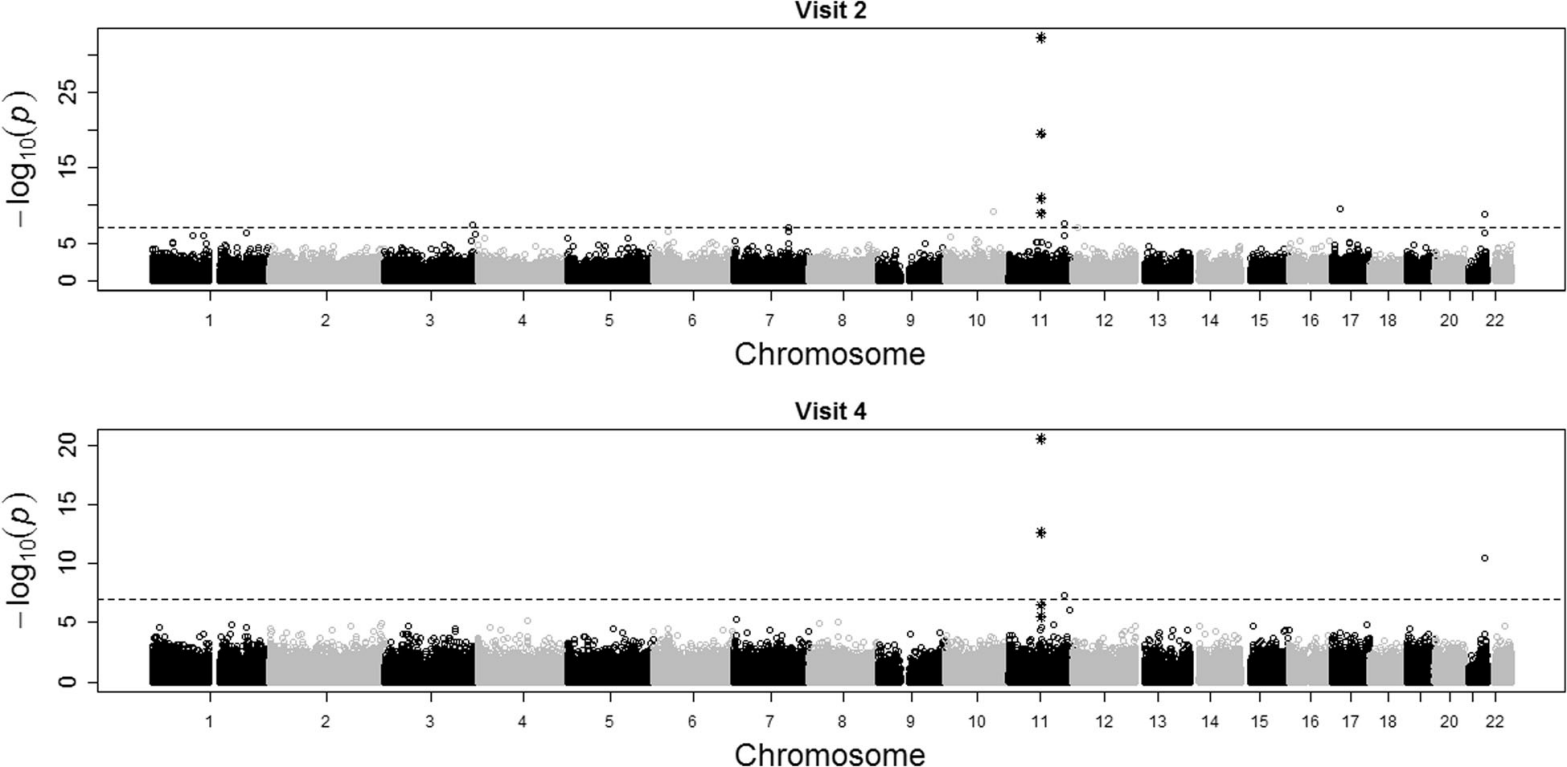
EWAS Manhattan plots for TG with age, center, sex, and 4 principal components as covariates using methylation data from visits 2 and 4. Stars indicate the 4 candidate CpGs

Figure 1 shows the EWAS results from the GAW20 real data at visits 2 and 4 and Table 1 shows the *p* values of the 4 CpGs found by Irvin et al. [3]. The Bonferroni corrected threshold is *p* = 1.08 × 10^− 7^, and at visit 2 and visit 4 there are 4 and 2 CpGs meeting this significance threshold, respectively. The differing sample sizes at visit 2 (995) and visit 4 (530) may contribute to these differences. The family structure was not accounted for in our analysis, but nevertheless, the test results were not unduly inflated (quantile–quantile [Q-Q] plots not shown), with genomic control inflation factors of 0.956 at visit 2 and 1.08 at visit 4.

**Table 1:** The 4 CpGs identified by Irvin et al. [3] and their *p* values from each EWAS on the GAW20 real data at visit 2 and visit 4

CpG	Chromosome	Position	Visit 2 *p* value	Visit 4 *p* value
cg00574958	11	68,607,622	6.11 × 10^− 33^	3.10 × 10^−21^
cg17058475	11	68,607,737	3.04 × 10^−20^	2.34 × 10^−13^
cg01082498	11	68,607,675	1.08 × 10^− 11^	3.16 × 10^− 6^
cg09737197	11	68,608,225	1.20 × 10^− 9^	3.27 × 10^− 7^

**Fig. 2:**
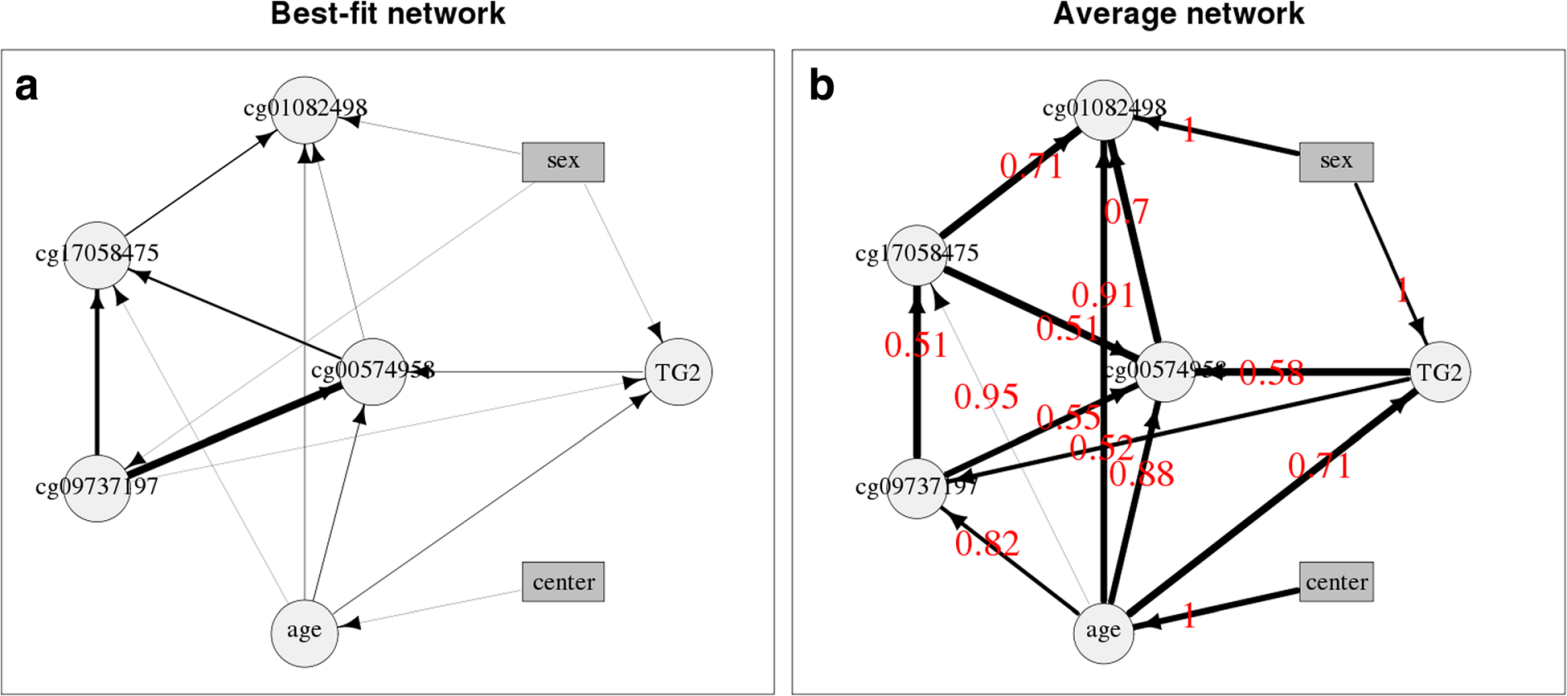
Networks of candidate CpGs in the GAW20 data at visit 4 together with variables for TGs, age, sex, and center. Circles and rectangles show continuous and discrete data respectively. **a** Best-fit Bayesian network. The thickness of the lines show the relative significance of the arrows. **b** Average Bayesian network. The thickness of the lines show the relative strength of the arrows; numbers in red show the (probability of) direction of the arrows

The best-fit Bayesian network shown in Fig. 2a shows connections between all the variables for the GAW20 real data at visit 2. In particular, the CpGs are strongly associated with one another, as would be expected, as they are close to one another on the epigenome and have similar EWAS results. Age and sex, as well as CpG cg09737197, are shown to directly influence TG level.

The average Bayesian network shown in Fig. 2b provides a better estimate of the direction of causality between variables. The line thickness of each arrow indicates the strength (probability) that the edge appears in the graph at all (in either direction), and the probability of the specified causal direction, given that the edge exists at all, is given by the number displayed in red on each arrow. Values near 0.5 show that the direction of causality is equally likely in either direction and may reflect correlation rather than implying causality. Although we may expect the CpGs to be associated with one another, we would not necessarily expect to be able to identify a causal relationship between them (given that no SNPs have been included as “genetic instruments”), and this is reflected in that most of the direction probabilities are close to 0.5 (specifically 0.51, 0.51, 0.52, and 0.55), although 0.7 and 0.71 between cg01082498 and two other CpGs is more indicative of a causal relationship than might be expected. Age has direction probabilities of 0.82, 0.88, 0.91, and 0.95 to the CpGs, suggesting a causal relationship, which is intuitive as age should affect methylation rather than vice versa. A possible argument that methylation could affect age is that the sample of individuals is biased with regard to methylation levels and age, for example, if individuals who are old are only sampled if they have particularly high methylation levels (for whatever reason). This would reflect causation in the sample rather than in the population. The direction of causality between methylation and TG level is not strong in either direction, with probabilities of 0.52 and 0.58 from CpGs to TG. Indeed, Sayols-Baixeras et al. [9] found evidence of causality between methylation and TGs going in either direction using the GAW20 data.

### Simulated data

**Fig. 3:**
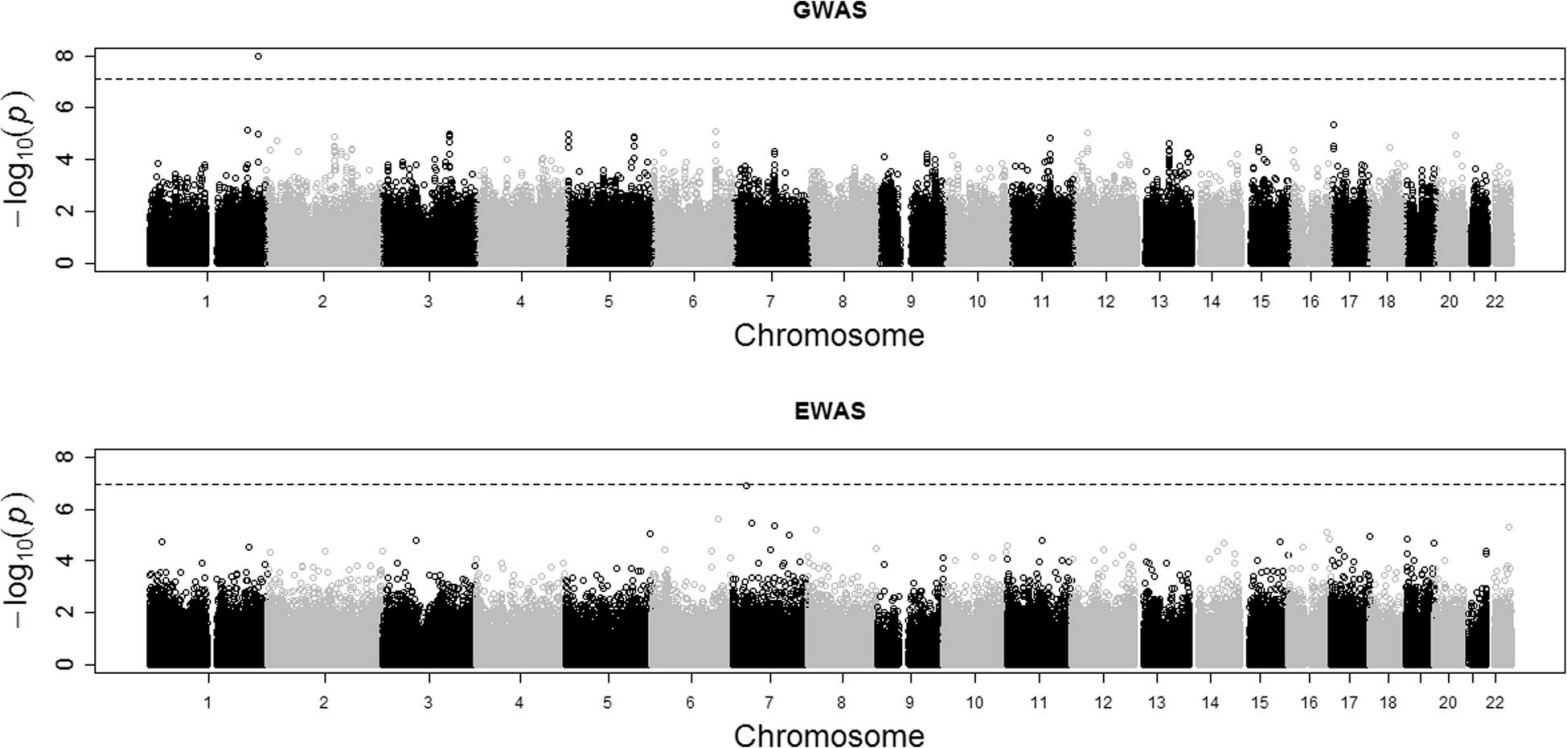
GWAS and EWAS of GAW20 simulated data for TG levels at visit 2, with age, sex, and center as covariates

Figure 3 shows plots of the results of the GWAS and EWAS. Q-Q plots of the results (not shown) did not show any signs of inflation with genomic control inflation factors of 1.004 for the GWAS and 0.996 for the EWAS. Only 1 SNP passed the Bonferroni corrected threshold for significance (*p* = 7.67 × 10^− 8^) and no CpGs were found to be significant from the EWAS. Table 2 shows the results for the 5 “known” causal SNPs and 5 corresponding CpGs together with their simulated theoretical expected heritabilities at stage 3 of the simulation, which, in the absence of any epigenetic effects, reflects the SNP effect sizes in relation to individual drug response. Given these relatively small effects, and that CpGs operate not through additional main effects but through modifying the effect of the corresponding SNP, it is perhaps not surprising that only 1 SNP and no CpGs were found to be significant. An alternative explanation could be the presence of unaccounted for confounding factors; however, the detailed documentation for the data simulation provided in the GAW “Answers” suggests that there were no additional confounding factors to be accounted for.

**Table 2:** The 5 SNPs and corresponding CpGs that were used to simulate change in TG levels between drug treatment in the GAW20 simulated data with their simulated theoretical expected heritabilities and their GWAS and EWAS *p* values

SNP	Chromosome	Position	Heritability	SNP *p* value	CpG	CpG *p* value
rs9661059	1	230,556,033	0.125	1.08 × 10^−8^	cg00000363	0.0766
rs736004	6	5,067,728	0.075	0.0164	cg10480950	0.1229
rs1012116	8	89,466,383	0.100	0.00125	cg18772399	0.7496
rs10828412	10	23,476,515	0.025	0.000690	cg00045910	0.8427
rs4399565	17	13,407,619	0.050	0.0123	cg01242676	0.9250

**Fig. 4:**
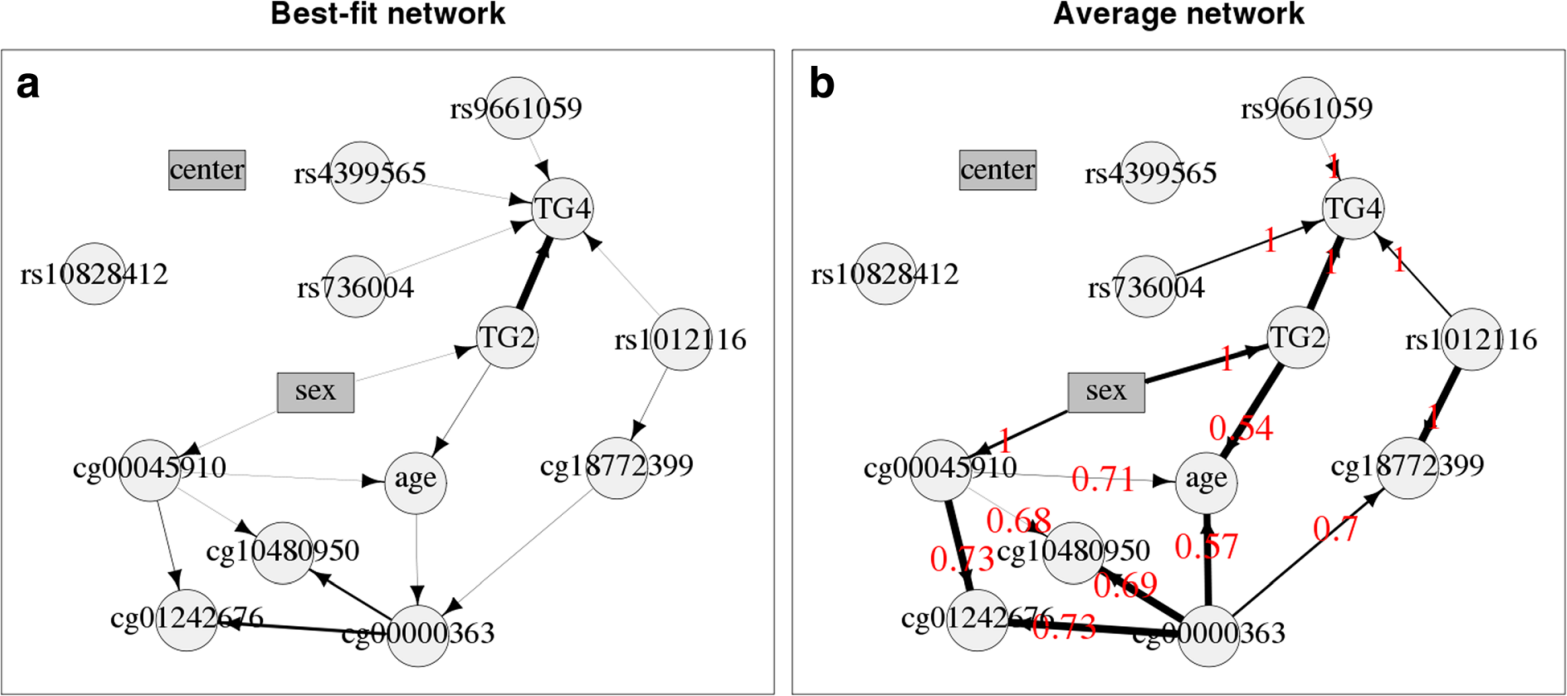
Networks of GAW20 simulated data of causal SNPs and CpGs at visit 4 together with variables for TGs, age, sex, and center. Circles and rectangles show continuous and discrete data respectively. **a** Best-fit Bayesian network. The thickness of the lines show the relative significance of the arrows. **b** Average Bayesian network. The thickness of the lines show the relative strength of the arrows; numbers in red show the (probability of) direction of the arrows

Figure 4a shows the best-fit Bayesian network and largely reflects the GWAS and EWAS results, such that most SNPs are related to a change in TG levels, but the CpGs are not. The only corresponding SNP and CpG connected to one another are rs1012116 and cg18772399. The CpGs are connected to one another, despite being randomly chosen across the epigenome on different chromosomes. This most probably reflects that different individuals tend to show similar levels of methylation across the whole epigenome, rather than any other interesting characteristics related to the drug-response simulation.

Figure 4b shows the average Bayesian network and very similar results to the best-fit network but with fewer arrows. After the strength threshold of 0.441 is applied to the average network, the arrow showing SNP rs4399565 relating to the change in TG levels is no longer plotted, highlighting the weak association. The strengths of edges (probability of a relationship going in either direction) from rs9661059, rs736004, rs1012116, rs10828412, and rs4399565 to the change in TG levels are 0.538, 0.654, 0.640, 0.394, and 0.441, respectively. It was suggested at the GAW20 workshop that, given the nature of the simulated data, variables for the interaction of SNPs and their corresponding CpG may give stronger associations with change in TG levels than are seen when modeling main effects of SNPs and CpGs. However, further investigation indicated that including such variables did not improve the levels of association detected (results not shown). The direction value of the arrows highlights the constraints, such that the arrow must always be in the shown direction if it is equal to 1. The direction value between CpGs are not too close to 1, showing there is not strong evidence for a causal relationship in one direction.

## Discussion

A simple EWAS of the GAW20 real data showed that the 4 CpGs previously detected by Irvin et al. [3] as associated with TGs, were also associated in the GAW20 real data. This association and the high correlation between CpGs resulted in a fitted Bayesian network that showed TG level to be dependent directly or indirectly on all the other variables.

The GAW20 simulated data presented more difficulties than the real data. From the GAW20 solutions it was known in advance that 5 SNPs and 5 corresponding CpGs were used to simulate change in TG. However, a simple GWAS and a simple EWAS only detected one of the SNPs. This can most probably be explained by the small effect sizes and small sample size of the data set, given that the 1 SNP detected had the largest effect size. Despite the complex nature of the simulated data and the weak association results, we did see some relationships between SNPs and a change in TG levels.

There are many benefits to the use of Bayesian networks. A particular benefit is the identification of previously overlooked possible causal relationships between variables in a biological system. Although not a rigorous test of causality, they form a useful additional technique to help direct further hypotheses about the system, as well as future studies and analyses. Visualization of Bayesian networks is a useful tool when there are many different variables operating within a system to aid the identification of interesting possible causal structures.

Bayesian networks do have some drawbacks, such as needing to search through a potentially large network space to find the best-fit network. The processing time for this can be improved by reducing the network space by imposing constraints between some variables and/or by the use of parallel computing. The optimality of the procedure can be improved with the use of random restarts and the development of different search algorithms.

## Conclusions

The GAW20 real data showed stronger associations between variables than the GAW20 simulated data, resulting in a better-connected, fitted Bayesian network. Despite some difficulties, Bayesian networks provide a further tool beyond detecting individual significant associations and may aid better understanding of biological systems to ultimately inform drug development.
